# Fat _max_ as an index of aerobic exercise performance in mice during uphill running

**DOI:** 10.1371/journal.pone.0193470

**Published:** 2018-02-23

**Authors:** Kengo Ishihara, Hirokazu Taniguchi

**Affiliations:** Faculty of Agriculture, Ryukoku University, Shiga, Japan; Max Delbruck Centrum fur Molekulare Medizin Berlin Buch, GERMANY

## Abstract

Endurance exercise performance has been used as a representative index in experimental animal models in the field of health sciences, exercise physiology, comparative physiology, food function or nutritional physiology. The objective of the present study was to evaluate the effectiveness of Fat_max_ (the exercise intensity that elicits maximal fat oxidation) as an additional index of endurance exercise performance that can be measured during running at submaximal exercise intensity in mice. We measured both Fat_max_ and Vo_2 peak_ of trained ICR mice that voluntary exercised for 8 weeks and compared them with a sedentary group of mice at multiple inclinations of 20, 30, 40, and 50° on a treadmill. The Vo_2_ at Fat_max_ of the training group was significantly higher than that of the sedentary group at inclinations of 30 and 40° (P < 0.001). The running speed at Fat_max_ of the training group was significantly higher than that of the sedentary group at inclinations of 20, 30, and 40° (P < 0.05). Blood lactate levels sharply increased in the sedentary group (7.33 ± 2.58 mM) compared to the training group (3.13 ± 1.00 mM, P < 0.01) when running speeds exceeded the Fat_max_ of sedentary mice. Vo_2_ at Fat_max_ significantly correlated to Vo_2 peak_, running time to fatigue, and lactic acid level during running (P < 0.05) although the reproducibility of Vo_2 peak_ was higher than that of Vo_2_ at Fat_max_. In conclusion, Fat_max_ can be used as a functional assessment of the endurance exercise performance of mice during submaximal exercise intensity.

## Introduction

Exercise has diverse health promotion effects. It is known that systemic endurance exercises are effective for the prevention of cardiovascular diseases. Since exercise intensity (watt, workload) and oxygen consumption (Vo_2_) are linearly associated, Vo_2 max_ is widely used as a reliable index of aerobic exercise performance in human study [1]. However, in rodent studies [2], the term "Vo_2 peak_" is used instead of "Vo_2 max_" because whether or not these animals exercise at their maximal intensity and whether the observed highest Vo_2_ reflects their maximal effort remains under active debate [3, 4]. To measure Vo_2 peak_ of mice, researchers motivate mice to run on a slope until they reach their maximum exercise intensity.

Petrosino et al. [5] reported a new method to measure aerobic exercise capacity during submaximal exercise. The performance indices during submaximal exercise have several advantages compared to those during maximal exercise for laboratory rodents to reduce their maximal effort [6].

In humans, aerobic exercise performance can be measured during submaximal exercises using various indices. Anaerobic threshold, ventilation threshold, or lactate threshold are generally used as indices of aerobic exercise performance during submaximal exercise in humans [7–9]. Estimation of ventilation threshold is inapplicable in rodents because their breath gas is collected by the open circuit method and therefore it is, in principle, impossible to measure the ventilation volume. Measurement of lactate threshold is not a practical method in rodents because collecting blood continuously from small animals during exercise requires the placement of a cannula.

Fat_max_ is an index of aerobic exercise performance that can be measured during submaximal exercise. Fat_max_ is the exercise intensity with peak fat oxidation [10–13]. The Fat_max_ of an active person is significantly higher than that of a sedentary person [11, 14, 15]. The reproducibility of Fat_max_ is still controversial [16, 17]. It is not known if Fat_max_ can be an indicator of aerobic exercise performance in experimental small animals.

Therefore, the major aim of the present study was to evaluate the effectiveness of Fat_max_ in detecting the improvements of aerobic exercise performance in mice. We measured both Fat_max_ and Vo_2 peak_ in trained mice that voluntarily exercised for 8 weeks and compared these variables with those of a sedentary group of mice.

## Materials and methods

### Ethics statement

All procedures were approved by the Animal Care and Use Committee of the Ryukoku University (No. 2015-3-1) and performed in accordance with the Animal Experimentation Guidelines of Ryukoku University.

### Apparatus

Running exercise was performed on a treadmill in a metabolic chamber, which included the single-lane test treadmill. The original material of the belt was rubber with irregularities for increasing friction. We coated the surface of the treadmill lane with a stretchable cloth in order to increase the friction and provide an anti-slip coating. Stainless steel grids at the end of the lines provided an electrical stimulus of 0.25 mA to keep the mice running.

### Animals and acclimatization

A total of 27 male ICR mice (Japan Shizuoka Laboratory Center, Hamamatsu, Japan) were housed in controlled conditions of temperature (24.5 ± 1°C), humidity (50 ± 5%), and lighting (lights on from 12:00 to 0:00). They were provided with a stock diet (D12450B; Research diet, NJ, USA) and water ad libitum. Strewment was changed every third day. The mice were acclimatized to the treadmill within a week from the start of breeding. Acclimatization consisted of three training sessions with 24 hours of recovery between sessions. During acclimatization, the mice were placed on a motionless treadmill for three minutes, after which the shock grid was activated. Next, the treadmill was engaged to a walking speed of five m/min for five minutes and progressively increased up to 10 m/min for a total duration of 15 minutes of exercise.

### Experiment 1. Exercise protocol of spontaneous running training

Twelve male ICR mice (8 wk old) were used after acclimation to treadmill running. Their Vo_2 peak_ was measured at an inclination of 40°, as described below, and mice were randomly divided into two groups with equal body weight, Vo_2 peak_, and Fat_max_ (Table 1). Six mice that formed the training group were housed individually with a spontaneous running saucer (Ware manufacturing, Inc., Phoenix, AZ, USA) for 8 weeks. The remaining six mice were housed without a running wheel for 8 weeks.

**Table 1:** Physiological parameters of training and sedentary group.

	Training	Sedentary
Body weight (g)	36.3 ± 3.4	38.6 ± 2.4
Vo_2 peak_ (mL/min/kg)	160.4 ± 10.7	157.0 ± 10.1
Vo_2_ at Fat_max_ (mL/min/kg)	155.3 ± 17.4	154.4 ± 7.49

Value are means ± SD (n = 6).

On 9th and 10th weeks, all mice ran four times, in random order, on the anti-slip coated lane at inclinations of 20, 30, 40, and 50°. Each running experiment was conducted at intervals of one day or more. Each mouse had a regular 10 min warm-up at each prescribed inclination, which was a 5-min running at 5 m/min followed by 5-min running at 10 m/min and the inclination was not changed until exhaustion. The treadmill velocity was then increased by 1 m/min every 30 seconds. Exhaustion (endpoint denoting time to stop the treadmill) was defined as the point at which the mice maintained continuous contact with the shock grid for five seconds or were unable to, or refused to run further [18]. Vo_2 peak_ and Fat_max_ were detected using respiratory gas measurement as described below.

On 11st week, all the mice were measured blood lactic acid concentration during resting and running at the submaximal exercise intensity as described below.

### Experiment 2. Exercise protocol of reproducibility test

Fifteen male ICR mice (6 wk old, body weight 32.1 ± 1.9 g) were used after acclimation to treadmill running. All mice ran two times, in random order, on the anti-slip coated lane at inclinations of 40°. The running speed of the treadmill was incremented as described in Experiment 1. Each running experiment was conducted at intervals of one day. Vo_2 peak_ and Fat_max_ were detected using respiratory gas measurement described below.

### Gas measurement

Ambient air was let into the treadmill chamber at a rate of 1.0 L/min. The air flowed from the front of the treadmill to the rear and then returned toward the front under the belt. This created a rapid, circular "loop" of mixed gases, from which a sample was drawn for analysis every 15 sec. Gas samples were extracted from the mass spectrometry gas analyzer (ALCO-2000, Chiba, Japan). The gas analyzers have a 2% measurement accuracy and were calibrated with standardized gas mixtures before every test session. ALCO2000 computer software collected gas concentration and flow to calculate the oxygen consumption (Vo_2_) and carbon dioxide expiration (Vco_2_) from the treadmill every 15 seconds. were calculated based on Frayn's equation [19].

$$\mathrm{Fat}\;\mathrm{oxidation}\;(g•\min^{‑1})=1.67\;V_{O}{}_{{}_{2}}(g•\min^{‑1})‑\;1.67\;V_{{\mathrm{CO}}_{2}}(g•\min^{‑1})$$

$$V_{O_{2}}=(F_{{\mathrm{EN}}_{2}}/F_{{\mathrm{IN}}_{2}}*F_{{\mathrm{IO}}_{2}}-F_{{\mathrm{EO}}_{2}})/100*\mathrm{Flow}*1000\;[\mathrm{mL}/\min.\;\mathrm{STPD}]$$

$$V_{{\mathrm{CO}}_{2}}=(F_{{\mathrm{ECO}}_{2}}-F_{{\mathrm{ICO}}_{2}})/100*\mathrm{Flow}*1000\;[\mathrm{mL}/\min.\;\mathrm{STPD}]$$

$$F_{E}**\;:\;\mathrm{concentration}\;\mathrm{of}\;\mathrm{exhaust}‑**[\%]$$

$$F_{I}**\;:\;\mathrm{concentration}\;\mathrm{of}\;\mathrm{supply}‑**[\%]$$

Flow:flowrate[L/min.STPD]

To allow rapid comparisons over a wide range of body weights (especially with human data), dimensional analyses and empirical studies have shown that Vo_2_ should be divided by the body mass raised to the power of 0.75 [4, 20, 21, 22]. Vo_2 peak_ was defined as the highest observed value of Vo_2_. Fat_max_ was defined as the exercise intensity that elicited the maximum fat oxidation.

### Measurement of blood lactate during submaximal exercise intensity of running

Blood lactic acid concentrations at rest and while running at two different intensities were compared in all mice. Each mouse ran as the same exercise protocol described in experiment 1 until the velocity reached 18 or 24 m/min at the slope of 40° on different days in random order. An exercise intensity of 18 m/min corresponded to an exercise intensity of Fat_max_ in the sedentary group. An exercise intensity of 24 m/min corresponded to an intermediate exercise intensity between Fat_max_ of sedentary (18 m/min) and training group (30 m/min). An exercise intensity of 18 and 24 m/min corresponded to 60 and 80% Fat_max_ in the training group, respectively. When running velocity reached 18 or 24 m/min, 0.7 μL of blood (via tail vein prick) was collected within 1 min, and was analyzed on a handheld lactate meter (Lactate pro-sensor 2, Arkray, Japan). The resting blood lactate concentration was measured on another day. Each running experiment was conducted at intervals of one day or more. For all testing, the same device was utilized to reduce variability.

### Statistical analysis

Values are expressed as means ± standard deviation (SD). Statistical analysis was carried out with one-way ANOVA, followed by Tukey’s post-hoc test for the comparisons between 20° and the other inclinations. Statistical analysis between the sedentary and training group was carried out with unpaired two-tailed t-test with each degree of inclination. Pearson’s product moment correlation analyses were used to examine bivariate relationships between index (Vo_2 peak_, Vo_2_ at Fat_max_, running time until fatigue, and plasma lactic acid level). The threshold for statistical significance was set to P < 0.05. All statistical analyses were performed using Prism software (version 7, GraphPad, CA, USA).

## Results

**Fig 1:**
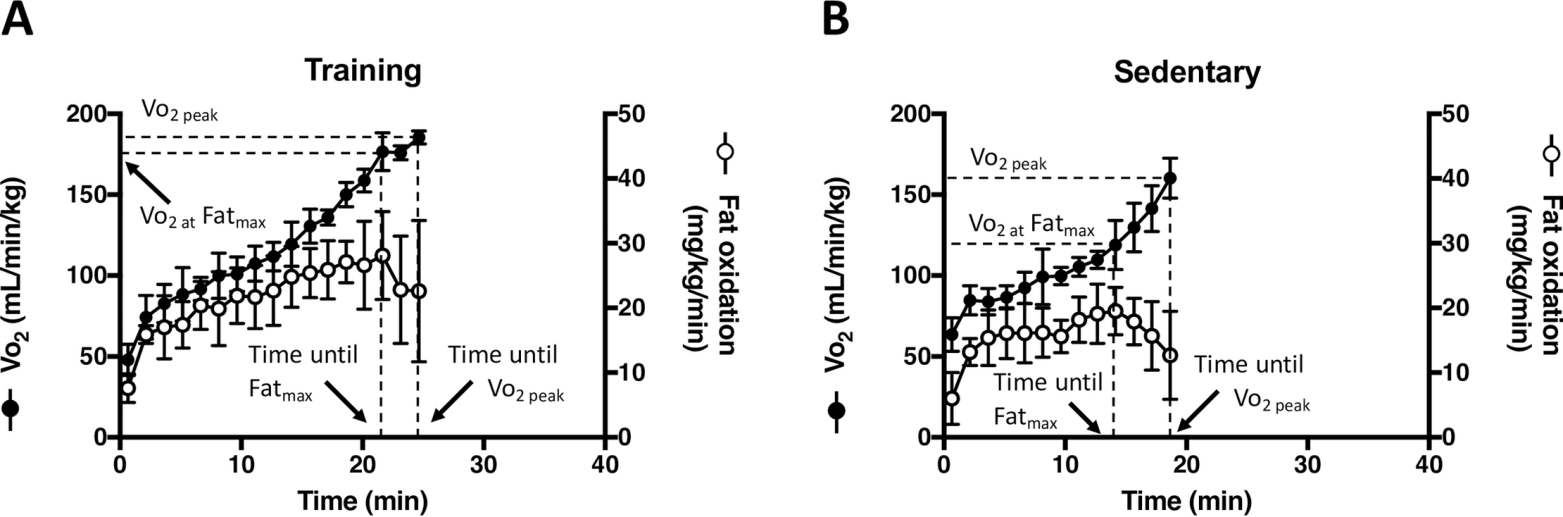
**Representative changes in Vo**_**2**_ **and fat oxidation while running and at inclinations of 40**° **of training (A) and sedentary (B) mice (n = 6).** Vo_2 peak_ is the maximum Vo_2_ observed while running. Fat_max_ is the exercise intensity that elicits maximum fat oxidation. Vo_2_ at Fat_max_ is the observed Vo_2_ at the exercise intensity of Fat_max_. The treadmill velocity was as follows: 0–5 min, 5 m/min; 5–10 min, 10 m/min; and then increased by 1 m/min every 30 seconds. Each running experiment at different inclinations was conducted at intervals of one day or more. Values are means ± SD (n = 6).

**Fig 1** shows the representative changes in Vo_2_ and fat oxidation in training and sedentary group during running. Vo_2_ continuously increased as the running speed increased and reached the highest value (Vo_2 peak_) in training and sedentary group. Based on the respiratory gas component while running, we calculated the fat oxidation of each mouse. Fat oxidation reached a peak and began to decline when exercise intensity exceeded the specific level for each mouse. We defined Fat_max_ of each mouse as the exercise intensity at which the fat oxidation reaches its maximum. We also defined time until Fat_max_ and time until Vo_2 peak_.

Fat_max_ could detect small improvements in endurance exercise performance due to voluntary running. The differences between Vo_2 peak_ and Vo_2_ at Fat_max_ were 44.6 and 13.6 mL/min/kg in sedentary and training mice, respectively. Therefore, we compared the indices of Vo_2 peak_ and Vo_2_ at Fat_max_, indices of time until Vo_2 peak_ and time until Fat_max_, and indices of speed at Vo_2 peak_ and speed at Fat_max_ between training and sedentary group in the following figures.

### Linear regression between Vo_2_ and running speed while hilly running

**Fig 2:**
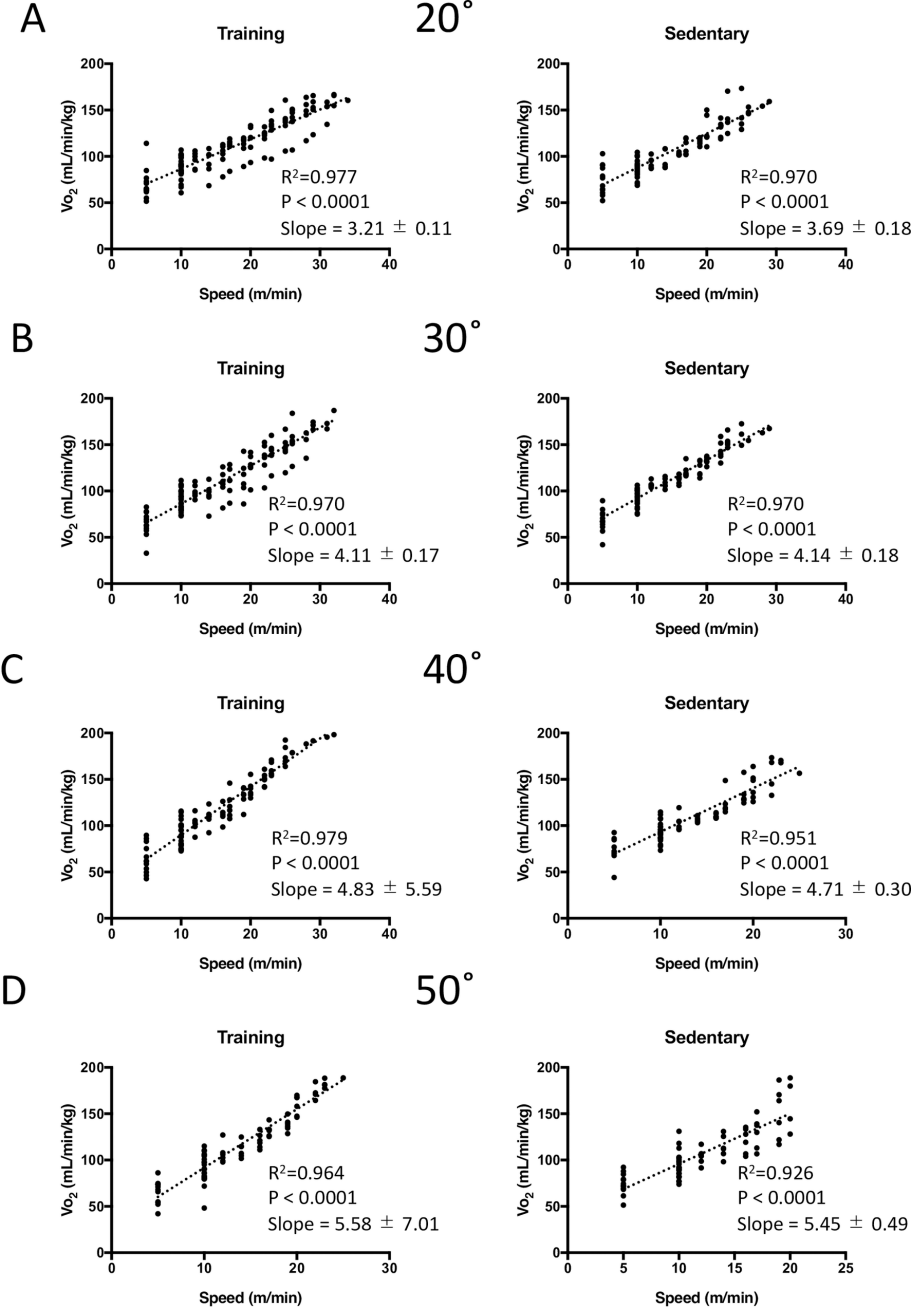
**Vo**_**2**_ **at increasing running speed while running at inclinations of 20 (A), 30 (B), 40 (C), and 50**° **(D) in training and sedentary mice (n = 6).** The trained mice were housed in cages with a running saucer for 8 weeks. The running protocol was described in Fig 1. Individual data are shown (n = 6).

**Fig 2** shows that Vo_2_ linear regression between Vo_2_ and running speed at all inclinations between 20 and 50° (experiment 1). The correlations between Vo_2_ and running speed were not less than 0.926 in all the inclination. The slope of linear regression curve between Vo_2_ and running speed was significantly higher in training group (5.58) compared to sedentary group (5.45) at the inclination of 50° (P < 0.01).

### Oxygen consumption at Fat_max_

**Fig 3:**
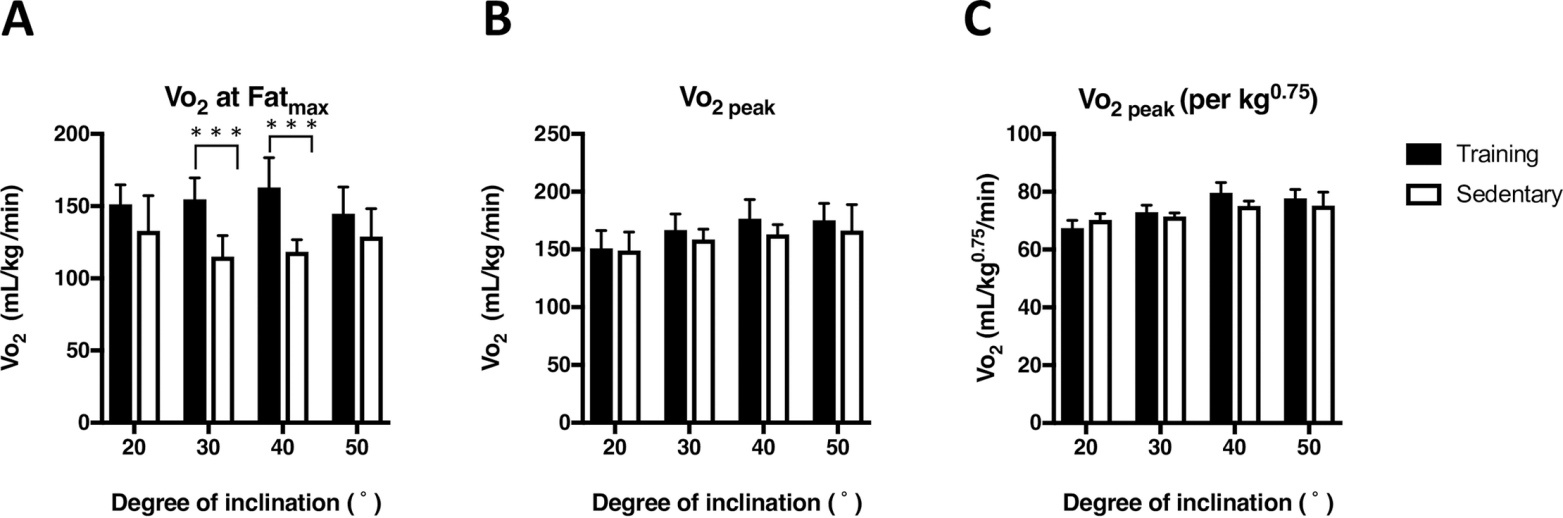
**Comparison between Vo**_**2**_ **at the exercise intensity that elicits maximum fat oxidation (Fat**_**max,**_ **A), Vo**_**2**_ **(B), and Vo**_**2**_ **normalized to the body mass raised to the power of 0.75 (C).** The running protocol was described in Fig 1. Values are mean ± SD (n = 6). *** P<0.001 between training and sedentary groups.

**Fig 3A** shows the Vo_2_ at the exercise intensity of Fat_max_ at each inclination (experiment 1). There observed significant differences in Vo_2_ at Fat_max_ between training and sedentary group (P < 0.001) at the inclination of 30 and 40°. The Vo_2_ at Fat_max_ in the training group measured at 20, 30, 40, and 50° inclinations were 151.3 ± 13.3, 154.8 ± 14.9, 162.9 ± 20.6, and 144.7 ± 18.5 mL/min/kg, respectively. Vo_2_ at Fat_max_ in the sedentary group measured at 20, 30, 40, and 50° inclinations were 132.9 ± 24.4, 115.1 ± 14.5, 118.5 ± 8.4, and 128.9 ± 19.4 mL/min/kg, respectively.

Vo_2 peak_ measured at each inclination were expressed as milliliters per kilogram per minute (**Fig 3B**) and expressed as milliliters per kilogram raised to the power of 0.75 per minute (**Fig 3C**). The Vo_2 peak_ of the training group measured at 20, 30, 40, and 50° inclination were 153.4 ± 12.1, 166.8 ± 14.0, 176.6 ± 16.6, and 175.3 ± 14.5 mL/min/kg, respectively. The Vo_2 peak_ of the sedentary group measured at 20, 30, 40, and 50° inclination were 156.1 ± 10.5, 158.7 ± 8.9, 163.1 ± 8.4, and 166.4 ± 22.5 mL/min/kg, respectively. There observed no significant differences in Vo_2_ peak between training and sedentary group at any inclinations.

### Running speed at Fat_max_

**Fig 4:**
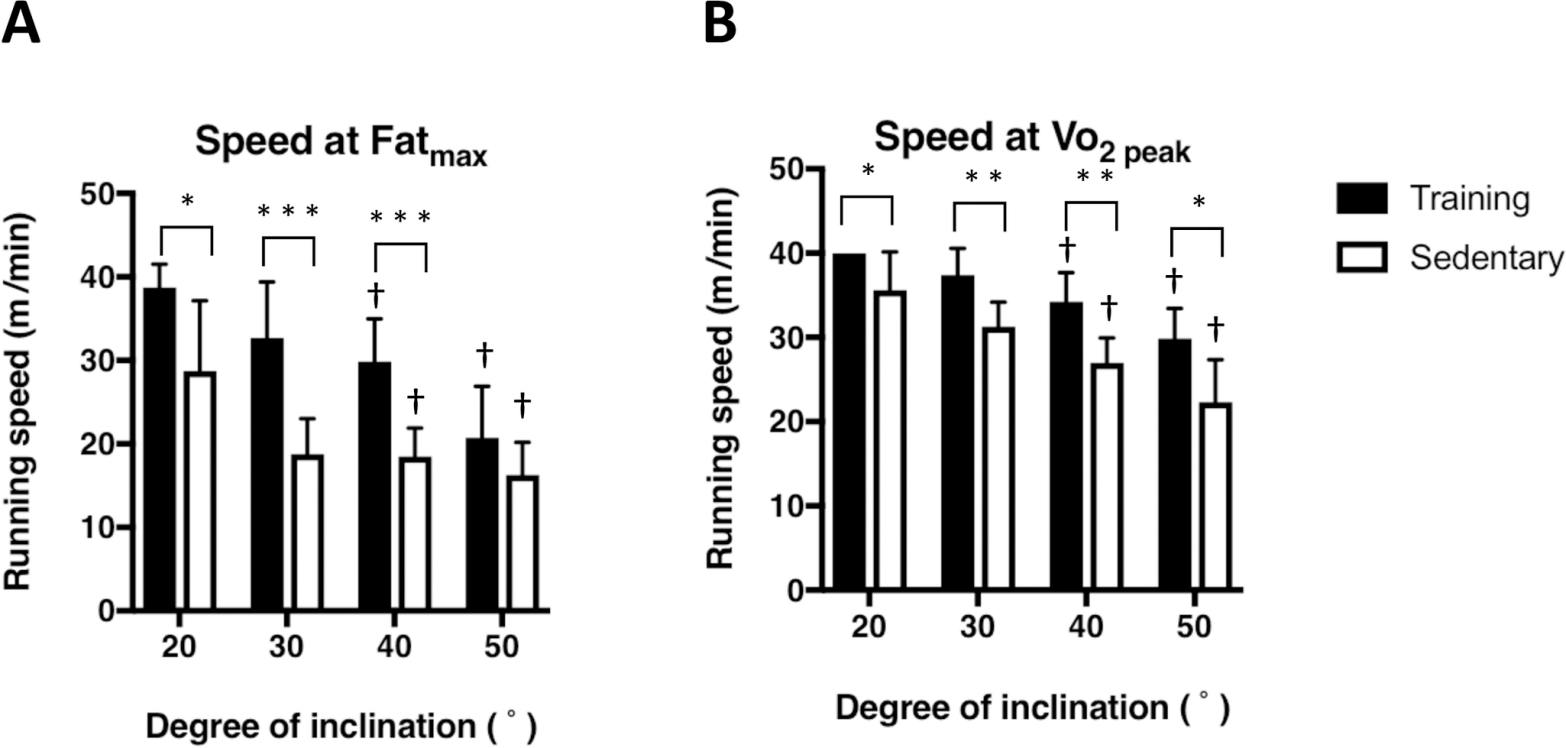
**Comparison between running speed at the exercise intensity that elicits maximum fat oxidation (Fat**_**max,**_ **A) and running speed** at Vo_2 peak_ **(B).** The running protocol was described in Fig 1. Values are mean ± SD (n = 6). *P < 0.05 compared as 0° of inclination. * P< 0.05, ** P<0.01, and *** P<0.001 between training and sedentary groups. † P < 0.05 compared to the corresponding value at 20°.

**Fig 4A** shows the running speeds at the Fat_max_ measured at each inclination (experiment 1). The running speeds at Fat_max_ of the training group were significantly higher than those of the sedentary group at inclinations of 20 (P < 0.05), 30 and 40° (P < 0.001). The running speeds at Fat_max_ of the training group measured at 20, 30, 40, and 50° inclinations were 38.7 ± 2.8, 32.7 ± 6.8, 29.9 ± 5.1, and 20.7 ± 6.2 m/min, respectively. The running speeds at Fat_max_ of the sedentary group measured at 20, 30, 40, and 50° inclinations were 28.7 ± 8.4, 18.8 ± 4.3, 18.5 ± 3.4, and 16.3 ± 3.9 m/min, respectively.

**Fig 4B** shows the running speed at Vo_2 peak_ at each inclination. The running speed at Vo_2 peak_ of the training group was significantly higher than that of the sedentary group at inclinations of 20, 30, 40, and 50° (P < 0.05). The running speeds at Vo_2 peak_ of the training group measured at 20, 30, 40, and 50° inclinations were 40.0 ± 0.1, 37.4 ± 3.2, 34.2 ± 3.5, and 29.9 ± 3.6 m/min, respectively. The running speeds at Vo_2 peak_ of the sedentary group measured at 20, 30, 40, and 50° inclinations were 39.0 ± 1.5, 31.3 ± 3.0, 27.0 ± 3.0, and 22.3 ± 5.0 m/min, respectively.

### Running time until Fat_max_

**Fig 5:**
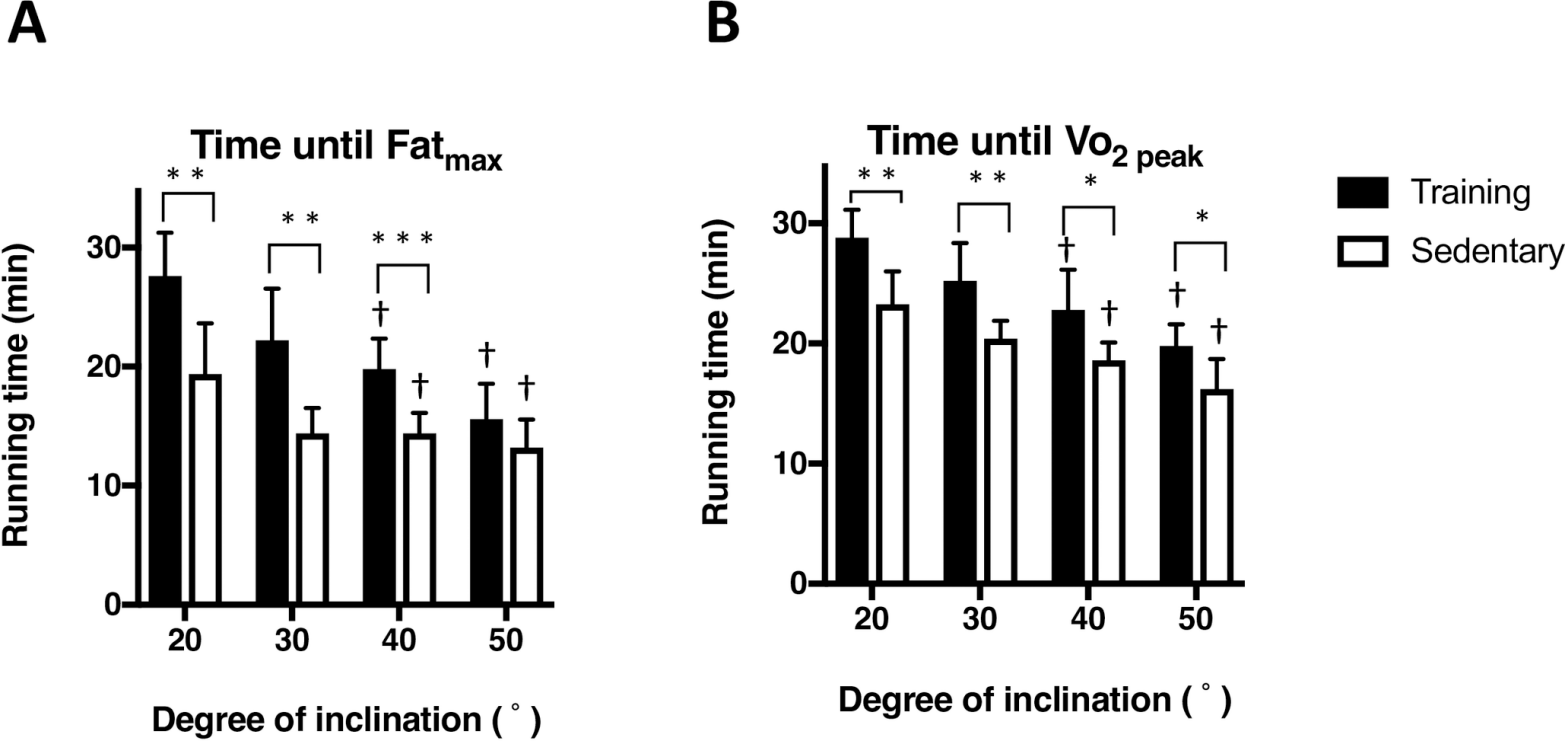
**Comparison between running time until the exercise intensity that elicits maximum fat oxidation (Fat**_**max**_**, A) and running time until** Vo_2 peak_ **(B).** The running protocol was described in Fig 1. Values are mean ± SD (n = 6). *P < 0.05, ** P<0.01, and *** P<0.001 between training and sedentary groups. † P < 0.05 compared to the corresponding value at 20°.

**Fig 5A** shows the running time until Fat_max_ measured at each inclination (experiment 1). The running time until Fat_max_ of the training group was significantly higher than that of the sedentary group at 20, 30 and 40° inclinations (P < 0.01). The running times until Fat_max_ of the training group measured at 20, 30, 40, and 50° inclinations were 27.6 ± 3.7, 22.2 ± 4.4, 19.8 ± 2.6, and 15.6 ± 3.0 min, respectively. The running times until Fat_max_ of the sedentary group measured at 20, 30, 40, and 50° were 19.4 ± 4.2, 14.4 ± 2.1, 14.4 ± 1.7, and 13.2 ± 2.3 min, respectively.

**Fig 5B** shows the running times until Vo_2 peak_ was measured at each inclination. The running time until Vo_2 peak_ of the training group was significantly higher than that of the sedentary group at inclinations of 20, 30, 40 and 50° (P < 0.05). The running time until Vo_2 peak_ of the training group measured at 20, 30, 40, and 50° inclinations were 28.8 ± 2.3, 25.2 ± 3.2, 22.8 ± 3.3, and 19.8 ± 1.8 min, respectively. The running times until Vo_2 peak_ of the sedentary group measured at 20, 30, 40, and 50° inclinations were 23.3 ± 2.7, 20.4 ± 1.5, 18.6 ± 1.5, and 16.2 ± 2.5 min, respectively.

### Blood lactate while running during submaximal running

**Fig 6:**
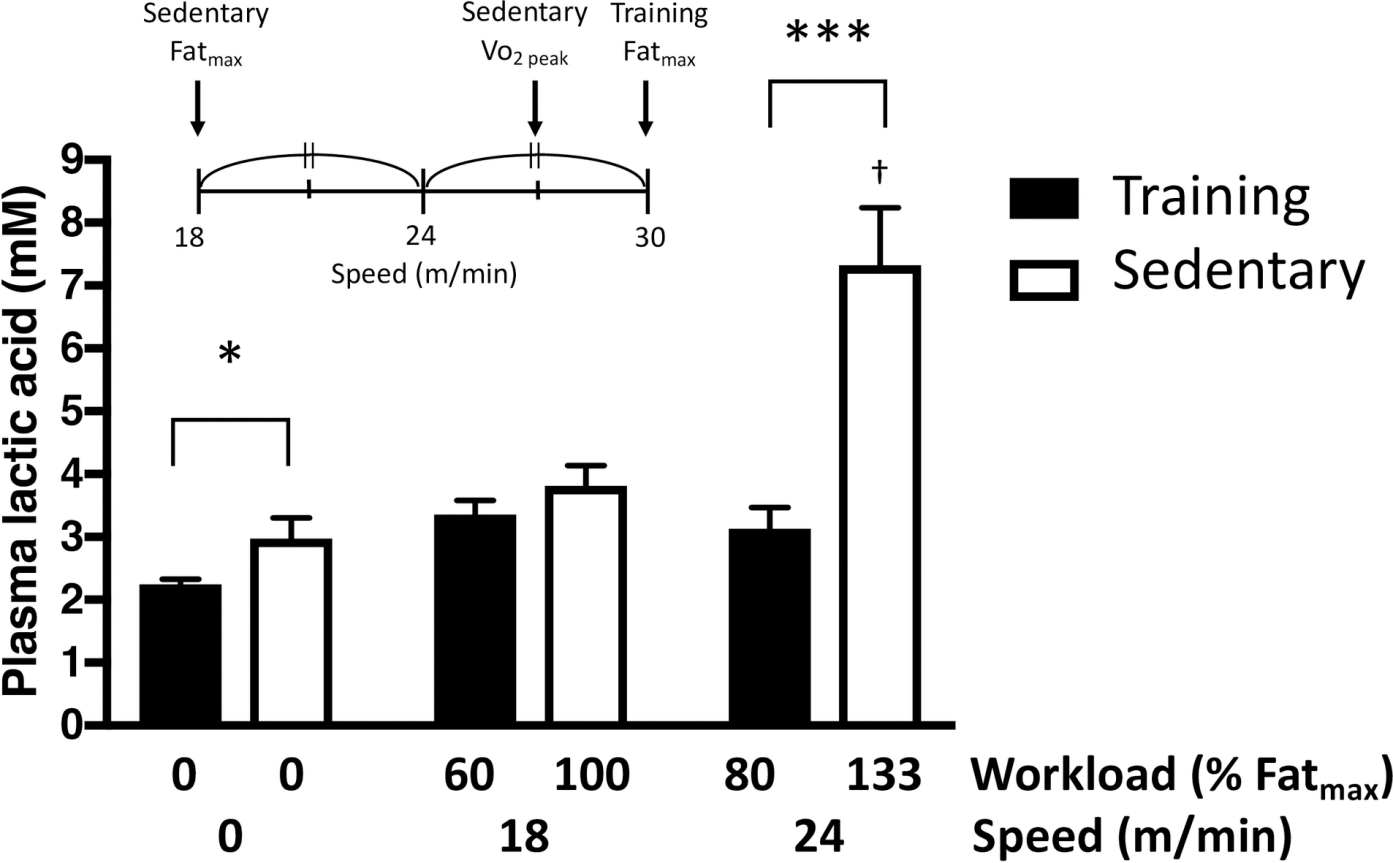
Blood lactate concentration during submaximal running. The running protocol was described in Fig 1. Running speed of 18 m/min corresponded to the Fat_max_ of sedentary group and running speed of 24 m/min corresponded to the half speed of Fat_max_ of sedentary and training group. Values are mean ± SD (n = 6). †P < 0.05 compared to the corresponding resting value. *P< 0.05, and ***P<0.001 between training and sedentary groups.

**Fig 6** shows the blood lactate concentration of the two groups during running at inclimation of 40° (experiment 1). The resting blood lactate concentration was 2.24 ± 0.26 and 2.98 ± 0.93 mM in the training and sedentary groups, respectively. While running at a speed of 18 m/min, which is the intensity of Fat_max_ in the sedentary group, blood lactate did not increase in both groups (3.36 ± 0.68 and 3.81 ± 0.91 mM in the training and sedentary groups, respectively). While running at a speed of 24 m/min, which is 133% intensity of Fat_max_ in the sedentary group and 80% intensity of Fat_max_ in the training group, blood lactate sharply increased and was significantly higher in the sedentary group (7.33±2.58 mM) than in the trainning group (3.13±1.00 mM, P < 0.001).

### Correlations among Vo_2_ at Fat_max,_ Vo2 _peak_, running time until fatigue, and plasma lactic acid concentration during running

**Fig 7:**
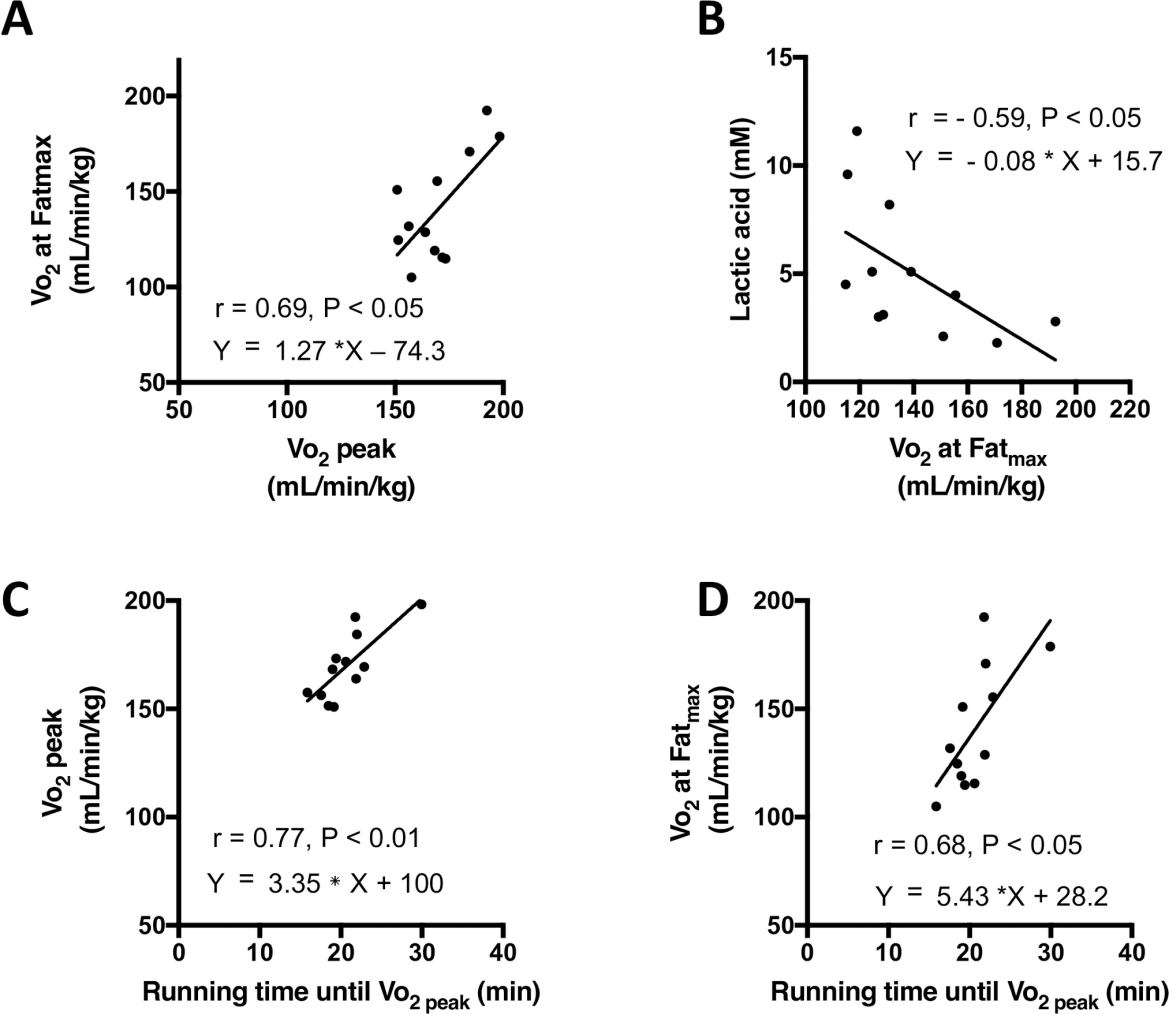
Correlations among Vo_2_ at Fat_max,_ Vo2 _peak_, running time until fatigue, and plasma lactic acid concentration during running at an inclination of 40°. The running protocol was described in Fig 1. Linear correlations were analyzed between Vo_2 peak_ and Vo_2_ at Fat_max_ (A), between Vo_2_ at Fat_max_ and plasma lactic acid concentration (B), between Vo_2 peak_ and running time until Vo_2 peak_ (C), and between Vo_2_ at Fat_max_ and running time until fatigue (D). Plasma lactic acid concentration was measured during running at a speed of 24 m/min at the inclination of 40° as described in Fig 6. The running protocol was described in Fig 1. Individual data are shown (n = 12).

**Fig 7** show the correlations among Vo_2_ at Fat_max,_ Vo_2 peak_, running time until Vo_2 peak_, and plasma lactic acid concentration during running at the inclination of 40° (experiment 1). Significant correlations were observed between Vo_2 peak_ and Vo_2_ at Fat_max_ (**A**, r = 0.69, P < 0.05), between Vo_2_ at Fat_max_ and plasma lactic acid concentration during running at the speed of 24 m/min (**B**, r = - 0.59, P < 0.05), between Vo_2 peak_ and running time until Vo2 _peak_ (**C**, r = 0.77, P < 0.01) and between Vo_2_ at Fat_max_ and running time until Vo2 _peak_ (**D,** r = 0.68, P < 0.05).

### Reproducibility of Vo_2_ at Fat_max_ during running at an inclination of 40°

**Fig 8:**
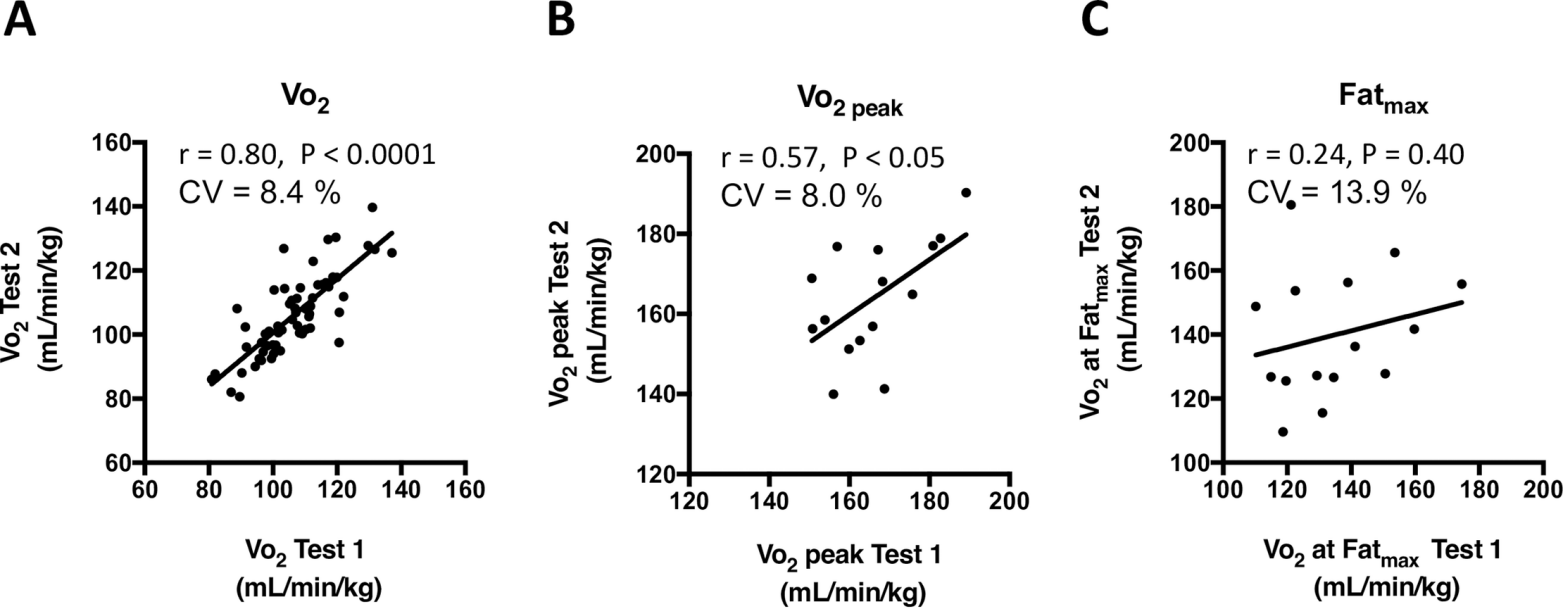
Reproducibility of Vo_2_ during submaximal running_,_ Vo_2 peak,_ and Vo_2_ at Fat_max_ during running at an inclination of 40°. Each mouse ran two times at intervals of one day. The running protocol is described in Fig 1. Linear correlations were analyzed between two tests. CV, coefficient of variation. Individual data are shown (n = 15).

**Fig 8** shows the reproducibility of Vo_2_ at submaximal exercise intensity, Vo_2 peak_ and Vo_2_ at Fat_max_ during exercise. Measurements of Vo_2 peak_ and Vo_2_ at Fat_max_ were conducted in fifteen mice at the inclination of 40° on 2 different days (experiment 2). **Fig 8A** shows the reproducibility of Vo_2_ during running at four submaximal velocities (5.7, 10.9, 12.7, and 14.4 m/min). Test-retest correlation of Vo_2_ was 0.80, and the coefficient of variation was 8.4%. **Fig 8B and 8C** shows the reproducibility for Vo_2 peak_ and Vo_2_ at Fat_max_ during exercise conducted at intervals of one day. Test-retest correlations of Vo_2 peak_ and Vo_2_ at Fat_max_ were 0.57 and 0.24, respectively. Test-retest coefficient of variations of Vo_2 peak_ and Vo_2_ at Fat_max_ were 8.0 and 13.9%, respectively.

## Discussion

The present study was designed to investigate whether Fat_max_, an index of endurance exercise performance in human, could detect the training effect of mice during submaximal exercise. Our main findings were that indices based on Fat_max_ 1) could detect small improvements in endurance exercise performance due to voluntary running, and 2) enabled the measurement of aerobic exercise performance during submaximal exercise with/without running at maximal speed.

The exercise protocol for optimum measurement of endurance exercise performance has been studied for various inclinations of treadmills. The exercise protocol by Kemi et al. [4, 20] is one of the most traditional protocols and a frequently quoted method [21–27]. In the method of Kemi et al., it was reported that the highest Vo_2 peak_ was observed with medium inclinations (15–35°). Ayachi et al. [3] reported that the Vo_2 peak_ observed in the incremental protocol at 25° inclination was the second highest and the highest Vo_2 peak_ was observed in the ramp protocol at 0° inclination in their study using one-year-old FVB mice. Petrosino et al. [5] investigated Vo_2 peak_ of mice at inclinations of 15°, which are less than inclination of 25° of Kemi protocol [4]. Therefore, in the present study, measurements of Fat_max_ were performed at various inclinations of 20, 30, 40 and 50° because sufficient experimental data have not been reported regarding these high inclinations.

An important and fundamental result in the study of Kemi et al. [20] was that a linear increase was observed in male and female rats and mice depending on the running speed at a middle (25°) inclination. The present study demonstrated that Vo_2_ linearly increased with the running velocity at the inclination of 20, 30, 40 and 50° in both training and sedentary group of mice (Fig 2), which indicated that running velocity corresponded to the exercise intensity in the present hilly running exercise protocol.

Another fundamental result in the study was the similarity of the measured values. Reported Vo_2 peak_ of forcefully trained mice for 8 weeks was 76.2 ± 4.2 mL/kg^0.75^・min in C57BL/6 mice [20] and was similar to our measured Vo_2 peak_ (79.6 ± 8.79 mL/kg^0.75^, Fig 3C) in ICR mice, which was larger than Vo_2 peak_ of 1-year old sedentary FVB/N mice (59.0 ± 0.61 mL/kg^0.75^・min, [3]).

In the preliminary examination, at an inclination of 40° or more, we observed that the mice slipped several times on the surface of the treadmill while running at high velocities. We coated the running belt of the treadmill with an anti-slip fabric to improve the friction with the sole of the foot. As a result of the modification, the slipping completely resolved even when the belt was wet with mouse urine (S1 Video). S1 Fig illustrates the effect of the anti-slip fabric coating, which significantly improved maximum running time until fatigue and tended to improve maximum running speed of mice compared to those without coating. Therefore, all of the studies were performed using treadmill coated with anti-slip fabric.

Fat oxidation increases with the exercise intensity but decreases when the exercise intensity exceeds the exercise intensity of Fat_max,_ Fat_max_ is the exercise intensity that elicits maximum fat oxidation and is the metabolic index that could be used to individualize training in healthy sedentary adults [28]. As shown in Fig 1, fat oxidation peaked at individually different running velocity in both training and sedentary group of mice during running.

One of the advantages of Fat _max_ is that these indices concerning Fat_max_ can be measured without any additional experiments. Another advantage is that it does not require loading maximum effort to mice as Fat _max_ can be measured during submaximal exercise. Apparatuses such as an electric grid or air jet have been used to motivate rodents to run until exhaustion in running exercise and difficulties in repeated endurance running tests with shock grid were reported [29] and an alternative to forced exercise assessment of murine exercise endurance without the use of a shock grid is proposed [6].

The reproducibility of Fat _max_ has been under debate. Reported intra-individual variability (coefficient of variation) of Fat_max_ values between 5 to 20% [11, 16, 17, 30, 31, 32]. The present study confirmed that the reproducibility of Fat _max_ (CV = 13.9%) was within the range of reported (Fig 8C). The reproducibility of Fat _max_ was lower than that of Vo_2 peak_ (CV = 8.0%). Significant correlations were observed between Vo_2_ at Fat_max_ and Vo_2 peak_ (r = 0.69, P < 0.05, Fig 7A), between Vo_2_ at Fat_max_ and running time until Vo_2 peak_ (r = 0.68, P < 0.05, Fig 8D) and between Vo_2_ at Fat_max_ and lactate acid concentration during submaximal exercise that corresponded to the half speed of Fat_max_ of sedentary and training group (r = -0.59, P < 0.05, Fig 7B).

Blood lactic acid significantly increased between 100 and 133% of Fat_max_ in sedentary group. Thus Fat_max_ was below intensity at lactate threshold and that probably lactate threshold is below 133% Fatmax, according to the data obtained with the sedentary group. The author should have measured blood lactate concentration during a steady state run at the Fat_max_ intensity of both group to consider the relationship between lactate threshold and the exercise intensity of Fat_max_ (Fig 6). Further research is required to establish an exercise protocol that can measure Fat_max_ with higher reproducibility and to consider whether 30 sec is sufficient to allow a steady state measurement of fat oxidation.

In conclusion, the present study showed that Fat_max_, an index of endurance exercise performance, could sensitively detect the effect of training in mice during submaximal running exercise at an inclination of 30 or 40°.

## Supporting information

S1 Fig Anti-slip fabric coating of the belt of treadmill enhanced maximum running time and speed at inclination of 40°. Male 20 wk old six ICR mice were run until fatigue on the treadmill with or without anti-slip fabric coating of the belt. Running experiments were conducted with crossover design and each mouse ran two times over 2 consecutive days. The treadmill velocity was as follows: 0–5 min, 5 m/min; 5–10 min, 10 m/min; and then increased by 1 m/min every 30 seconds until a maximum speed of 40 m/min was reached. Maximum running time until fatigue (A) and maximum running speed (B) were recorded. Values are mean ± SD (n = 6). *P < 0.05.
(TIF)

S1 Video Effect of anti-slip coating of the treadmill belt on running form at an inclination of 40°. At an inclination of 40° or more, we observed several episodes of slipping (right mouse) on the surface of the treadmill during running at high velocity. We coated the running belt of the treadmill with an anti-slip fabric to improve the friction with the sole of the foot, and as a result of the modification, the slip of the mouse completely disappears (left mouse).
(MOV)
