# Supplementary figures and images for: Fat max as an index of aerobic exercise performance in mice during uphill running
Source: PLoS One. 2018 Feb 23;13(2):e0193470. doi: 10.1371/journal.pone.0193470 (PMC5825145; doi:10.1371/journal.pone.0193470)

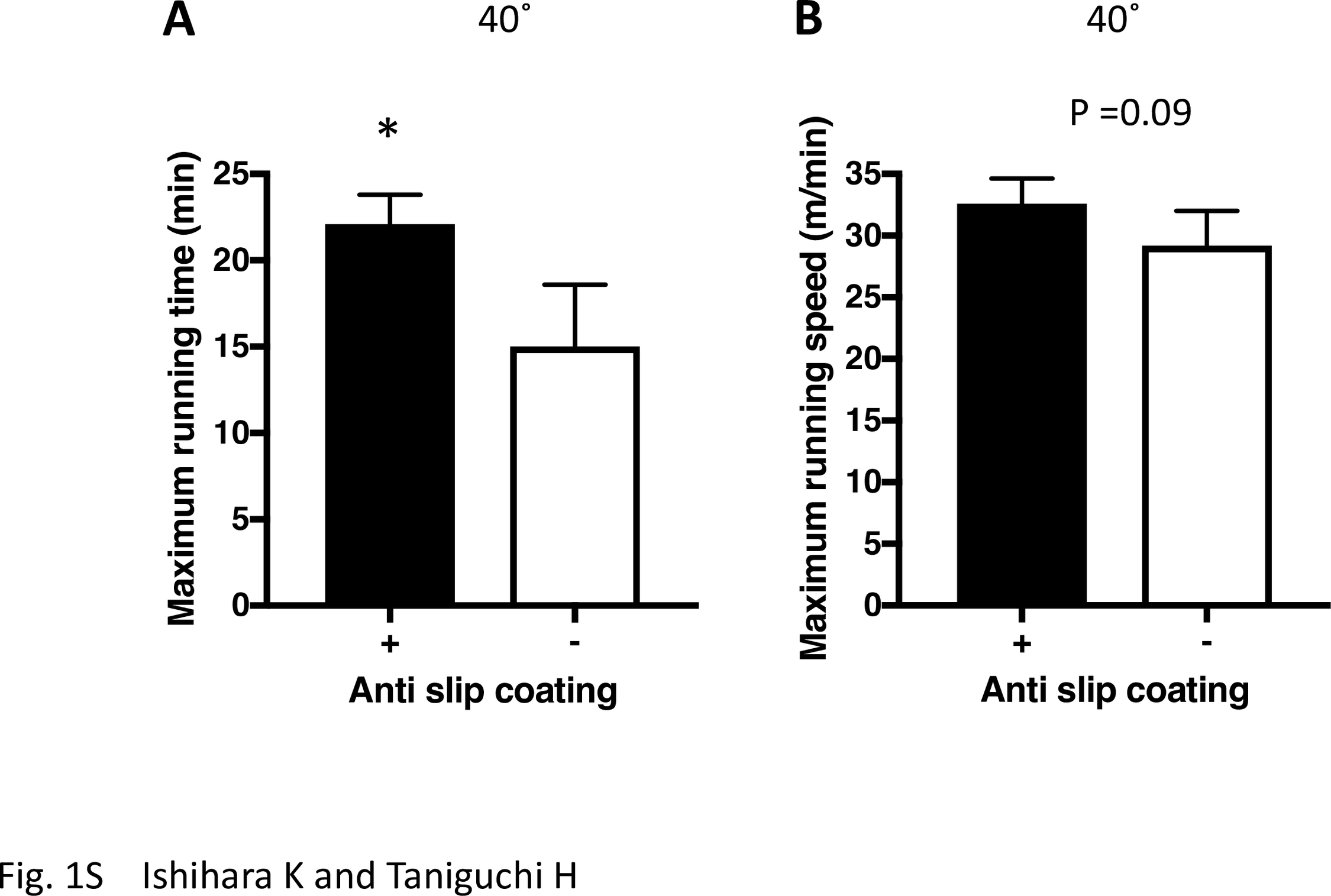

Supplement: S1 Fig — Male 20 wk old six ICR mice were run until fatigue on the treadmill with or without anti-slip fabric coating of the belt. Running experiments were conducted with crossover design and each mouse ran two times over 2 consecutive days. The treadmill velocity was as follows: 0–5 min, 5 m/min; 5–10 min, 10 m/min; and then increased by 1 m/min every 30 seconds until a maximum speed of 40 m/min was reached. Maximum running time until fatigue (A) and maximum running speed (B) were recorded. Values are mean ± SD (n = 6). *P < 0.05. (TIF) [file pone.0193470.s001.tif]
